# Tuberculosis examination using whole blood interferon-gamma release assay among health care workers in a Japanese hospital without tuberculosis-specific wards

**DOI:** 10.1186/2193-1801-2-440

**Published:** 2013-09-05

**Authors:** Eisuke Adachi, Mika Kogayu, Takeshi Fujii, Hiroshi Mae, Shoichi Shimizu, Yuichi Iwai, Hiroko Shibata, Masato Suzuki, Kohzoh Imai, Tomohiko Koibuchi

**Affiliations:** Department of Infectious Diseases and Applied Immunology, IMSUT Hospital of The Institute of Medical Science, The University of Tokyo, 4-6-1 Shirokanedai, Minato-ku, Tokyo, 108-8639 Japan; Department of Nursing, IMSUT Hospital of The Institute of Medical Science, The University of Tokyo, 4-6-1 Shirokanedai, Minato-ku, Tokyo, 108-8639 Japan; Department of Pharmacy, IMSUT Hospital of The Institute of Medical Science, The University of Tokyo, 4-6-1 Shirokanedai, Minato-ku, Tokyo, 108-8639 Japan; Department of Laboratory Medicine, IMSUT Hospital of The Institute of Medical Science, The University of Tokyo, 4-6-1 Shirokanedai, Minato-ku, Tokyo, 108-8639 Japan; Director, IMSUT Hospital of The Institute of Medical Science, The University of Tokyo, 4-6-1 Shirokanedai, Minato-ku, Tokyo, 108-8639 Japan

**Keywords:** Latent tuberculosis infection, Quantiferon-TB gold in tube, Latent tuberculosis infection baseline screening, Health care workers

## Abstract

Occupational latent tuberculosis infection (LTBI) among health care workers (HCWs) is an important public health issue. The objective of this study is to assess prevalence and risk factors of LTBI among Japanese HCWs by Quantiferon-TB Gold in Tube (QFT-GIT) and the structured questionnaire. This is a cross-sectional study involving HCWs from a hospital without tuberculosis-specific wards, receiving QFT-GIT for LTBI screening. We reviewed medical records of HCWs and questioned HCWs about exposure to *M*. *tuberculosis* and employment length in health care industries. 165 HCWs, approximately 80% of the total hospital staff, were enrolled in this study.18 out of 165 subjects had positive results, suggesting LTBI prevalence rate of 11%. Multiple regression analysis revealed a significant association between the positive or intermediate QFT-GIT results and history of contact investigation for tuberculosis**.** QFT-GIT positivity rate among HCWs is higher than among general population in Japan.

## Background

Occupational latent tuberculosis infection (LTBI) among health care workers (HCWs) is an important public health issue. World Health Organization (WHO) estimate of tuberculosis incidence of Japan in 2011 was 20 per 100,000 population per year. The Tuberculosis Surveillance Center of The research institute of Tuberculosis, Japan Anti-tuberculosis Association reported the incidence of tuberculosis of Japan was 18.2 in 2010 and 17.7 in 2011 (Rates per 100,000) and had continued to decline. However, approximately 80% of nurses with tuberculosis were speculated to be infected by nosocomial infection in Japan (Ohmori et al. 2007). The screening for LTBI by means of the tuberculin skin test (TST) or whole blood interferon-gamma release assays (IGRAs) and the administration of chemoprophylaxsis have been shown to be effective for prevention to develop active tuberculosis (Ringshausen et al. 2012; Pai et al. 2004). In Japan, TST use is limited for screening of LTBI, because most Japanese residents have received bacille Calmette-Guérin (BCG) vaccination on multiple occasions. Accordingly, The Japanese Society of Tuberculosis recommend the Quantiferon®-TB Gold (QFT-G) test for the screening of *Mycobacterium tuberculosis* (*M*. *tuberculosis*) infection for HCWs or health care students (Hotta et al. 2007).

Most of the previous studies described the prevalence of LTBI among Japanese HCWs were performed in tuberculosis referral hospitals. The objective of this study is to assess prevalence and risk factors of LTBI among Japanese HCWs by Quantiferon-TB Gold in Tube (QFT-GIT), a new version of QFT-G, and the structured questionnaire in a hospital without tuberculosis-specific wards.

## Methods

### Setting and design

This is a cross-sectional study involving HCWs at the hospital affiliated with the Institute of Medical Science, the University of Tokyo (IMSUT hospital), receiving QFT-GIT for LTBI screening between November 2011 and July 2012. IMSUT Hospital has 135 beds and four negative pressure isolation rooms for patients diagnosed with active tuberculosis accidentally. The hospital is not a tuberculosis referral hospital and has no tuberculosis-specific wards. There were three active tuberculosis patients admitted in the IMSUT hospital in 2007. This is the highest number of annul admission of active tuberculosis cases in the hospital. We reviewed medical records of HCWs and questioned HCWs about exposure to *M*. *tuberculosis* and employment length in health care industries. We utilized the questionnaire consisting of the following questions: age, job category, history of contact investigation for tuberculosis, history of living with families who developed active tuberculosis, past history of active/latent tuberculosis infection, length of working in health care industries (general hospitals, tuberculosis referral hospitals, clinics, nursing home), history of working in tuberculosis ward/out patient department (OPD). Eligible subjects had been working as HCWs for more than 6 months and never had received IGRAs for LTBI screening. We excluded the HCWs with history of active tuberculosis. Written informed consent was obtained from subjects before completing the study questionnaire. This study was approved by the Research Ethics Committee, Institutional Review Board of IMSUT (accession number: 24-6, 24-32).

### Quantiferon®-TB Gold in Tube

Blood samples for QFT-GIT tests (Cellestis Limited. Australia) were collected at our hospital and QFT-GIT tests were performed at laboratories in the clinical laboratory testing industry (SRL Inc. Japan). Interferon-gamma (IFN-γ) responses to antigens that are at least 0.35 IU/mL greater than the nil control value are considered *M*. *tuberculosis* infection (“positive”). We also evaluated a prevalence using the value of 0.10 IU/mL as an alternative cut-off (“intermediate”) as the guideline of committees for the preventions of The Japanese Society of Tuberculosis. If the response is less than a cut-off value and their response to the mitogen positive control is greater than 0.5 IU/mL, the test result is considered negative.

### Statistical analysis

Comparisons of proportions were analyzed by the chi-square test or the Fisher exact test and a linear trend in proportions of ordered multiple groups were analyzed by Mantel-extension test. Multiple logistic regression analysis was performed (using JMP 9 software; SAS Institute Inc.) with the following independent variables: male/female, age groups, job category, history of contact investigation for tuberculosis, history of living with families who developed active tuberculosis and history of working in tuberculosis ward or OPD for more than 2 months.

## Results

165 HCWs, approximately 80% of the total hospital staff, were enrolled in this study. All subjects were Japanese. Table 1 shows the QFT-GIT positivity rates and characteristics of HCWs. 18 out of 165 subjects (10.9%) had positive results (0.35 IU/mL, cut-off). None of them manifested signs of active tuberculosis by their chest radiograph. 38 subjects (23%) had the value of QFT-GIT showed greater than 0.10 IU/mL. Notably, among HCWs aged 30-39 years, 14% of subjects (10/70) had positive QFT-GIT result. Table 2 shows the results of univariate analysis of tuberculosis infection risk factors. Univariate analysis revealed a significant association between the positive or intermediate QFT-GIT results and history of contact investigation for tuberculosis (odds ratio (OR) 3.4, 95% confidence interval (CI) 1.1 to 10.0). Neither age nor length of working in health care industries was associated with results of QFT-GIT among all HCWs. Among nurses and doctors aged 20-49 years, the percentage of HCWs with positive QFT-GIT results increased for each decade of age (odds ratios relative to persons aged 20-29 years, 2.5 (30-39 years), 2.9 (40-49 years)), but it did not reach statistical significance. Multiple logistic regression analysis to risk factors revealed that positive QFT-GIT result was significantly associated with male/female, the history of contact investigation for tuberculosis (Table 3).

**Table 1:** **Characteristics of HCWs and Quantiferon TB-Gold in tube positivity rate**

Total (number)		165
Gender	male	60 (36.4%)
	female	105 (63.4%)
Age (years, (IQR))		38^a^ (32 - 46)
Job category	nurse	72 (43.6%)
	doctor	53 (32.1%)
	laboratory*	12 (7.27%)
	pharmacist	12 (7.27%)
	other**	16 (9.70%)
Length of working in health care industries (years, (IQR))		13^a^ (7 - 18)
History of contact investigation for TB		25 (15.2%)
History of living with families who developed active TB		7 (4.24%)
History of working in TB ward/OPD		14 (8.48%)
QFT-GIT positivity (%)	Cut off: 0.35 IU/mL	18 (10.9%)
	Cut off: 0.10 IU/mL	38 (23.0%)

Note. *IQR*, interquartile range; *TB*, tuberculosis; *OPD*, outpatient department; *QFT*-*GIT*, Quantiferon TB-Gold in tube; * Laboratory technician ** radiography technicians, nutritionist, clerical, nursing assistants and social workers; ^a^median.

**Table 2:** **Association between results of Quantiferon TB-Gold in tube and risk factors for tuberculosis infection**

	No of subjects				No of subjects			
	Cut off: 0.35 IU/mL				Cut off: 0.1 IU/mL			
	≥ 0.35	≤ 0.35	OR(95% CI)		≥ 0.1	≤ 0.1	OR(95% CI)	
Male	10	50	2.4 (0.90 - 6.5, P = 0.12^b^)		15	45	1.2 (0.56 - 2.5, P = 0.7^b^)	
Female	8	97			23	82		
Job category								
Nurse	6	66	1.0^a^	P = 0.18^b^	14	58	1.0^a^	P = 0.69^b^
Doctor	5	48	1.1		12	41	1.2	
Laboratory*	3	9	3.7		4	8	2.1	
Pharmacist	3	9	3.7		3	9	1.4	
other**	1	15	0.73		5	11	1.9	
age								
20-29	2	24	1.0^a^	P = 0.76^b^	6	20	1.0^a^	P = 0.63^b^
30-39	10	60	2.0		17	53	1.1	
40-49	4	38	1.3		7	35	0.67	
50-	2	25	0.96		8	19	1.4	
Length of working in health care industries								
0-4	3	25	1.0^a^	P = 0.21^b^	6	22	1.0^a^	P = 0.87^b^
5-9	1	30	0.28		5	26	0.71	
10-14	6	37	1.4		11	32	1.3	
15-19	6	23	2.3		7	22	1.2	
20-	2	32	0.52		9	25	1.3	
History of contact investigation for TB								
Yes	6	19	3.4 (1.1 - 10.0, P = 0.034^b^)		12	13	4.0 (1.66 - 9.9, P = 0.003^b^)	
No	12	128			26	114		
History of living with families who developed active TB								
Yes	2	5	3.6 (0.64 - 20, P = 0.17^b^ )		3	4	2.6 (0.56 - 12.0, P = 0.20^b^)	
No	16	142			35	123		
History of working in TB ward/OPD								
Yes	1	13	0.61 (0.075 - 4.9, P = 1.0^b^)		3	11	0.90 (0.24 - 3.4, P = 1.0^b^)	
No	17	134			35	116		

Note. *OR*, odds ratio; *CI*, confidence interval; *TB*, tuberculosis; *OPD*, outpatient department; * Laboratory technician ** radiography technicians, nutritionist, clerical, nursing assistants and social workers; ^a^reference; ^b^Fisher Exact test.

**Table 3:** **Multivariate analysis of association between results of Quantiferon TB-Gold in tube and risk factors for tuberculosis infection**

	Cut off: 0.35 IU/mL		Cut off: 0.1 IU/mL	
	OR	P	OR	P
Male/ female	8.0	0.011	1.3	0.41
Job category				
Nurse	1.0^a^		1.0^a^	
Doctor	0.34	0.30	1.4	0.66
Laboratory technician	6.5	0.092	2.6	0.26
Pharmacist	4.9	0.096	1.7	0.51
Other*	1.5	0.76	2.9	0.13
Age				
20-29	1.0^a^		1.0^a^	
30-39	2.0	0.42	0.90	0.87
40-49	0.92	0.93	0.33	0.12
50-	0.23	0.26	0.83	0.78
History of contact investigation for TB	6.4	0.0077	4.7	0.0017
History of living with familes who developed active TB	6.0	0.11	3.3	0.24
History of working in TB ward/OPD	0.62	0.70	0.67	0.62

Note. *OR*, odds ratio; *TB*, tuberculosis; *OPD*, outpatient department * radiography technicians, nutritionist, clerical, nursing assistants and social workers; ^a^reference.

## Discussion

The utility of IGRAs in countries where BCG vaccine is widely used is supported by many studies (Pai et al. 2004). Harada et al. reported that a prevalence of LTBI among HCWs in a Japanese tuberculosis referral hospital was 9.9% by means of QFT-G (Harada et al. 2006). However, QFT-GIT, a new version of QFT-G, has higher sensitivity than QFT-G with high specificity for *M*. *tuberculosis* infection and is the currently recommended method for screening of LTBI in HCWs (Harada et al. 2008). Mori et al. reported that the QFT-G positivity rate of general population was 7.1% (40–69 years); it increased with age from 3.1% for the 40–49 years age group to 9.8% for those aged 60–69 (Mori et al. 2007).

We conducted here a cross-sectional study for HCWs receiving QFT-GIT. It showed the prevalence of LTBI to be 11%, which is remarkably higher than that among the general population (Mori et al. 2007). The QFT-GIT positivity rate in our study is consistent with the previous report from a tuberculosis referral hospital in Japan (Harada et al. 2006). We found that the prevalence of LTBI among HCWs in general hospitals without tubercu-losis wards and among HCWs in tuberculosis referral hospitals is similar. The report from the tuberculosis referral hospital also described that the positive QFT-G results were closely associated with age and with a history of working in a tuberculosis ward or an OPD of a tuberculosis clinic (Harada et al. 2006). In our study, positive QFT-GIT results were associated with the history of contact investigation for tuberculosis, that is suggestive of the history of exposure to *M*. *tuberculosis*. Of note, a high proportion of HCWs with positive/intermediate QFT-GIT results was laboratory technicians, which was consistent with the previous report (Harada et al. 2006). These suggest laboratory technicians are likely to be exposed to infectious agents in handling patients’ samples, such as sputum. There were no laboratory technicians with the history of contact investigation for tuberculosis in our study. We would like to offer that laboratory technicians should be included as subjects in performing contact investigation.

Our study has several limitations, including the small sample size and a single-center study. Diagnosis of LTBI by means of single QFT-GIT test remains controversial in accuracy and reliability, because the specific IFN-γ response may wane considerably with time after infection and a substantial technical variability exists (Ringshausen et al. 2012; Mori et al. 2007; Pai and O’Brien 2007). Mori et al. demonstrated that the probability of infection increases almost exponentially after 30 years of age (Mori 2000). The fact that age was not associated with results of QFT-GIT in our study may be due to the sample-size bias.

Our study is first to offer QFT-GIT positivity rate among Japanese HCWs in a hospital without tuberculosis-specific wards. To establish the evaluation of the LTBI by means of QFT-GIT test, several large, multi-center studies might be warranted.

## Conclusions

QFT-GIT positivity rate among HCWs is higher than among general population in Japan. This study showed the prevalence of LTBI among Japanese HCWs was 11%. Occupational LTBI among HCWs is an important public health issue.
